# Tombusvirus-yeast interactions identify conserved cell-intrinsic viral restriction factors

**DOI:** 10.3389/fpls.2014.00383

**Published:** 2014-08-11

**Authors:** Zsuzsanna Sasvari, Paulina Alatriste Gonzalez, Peter D. Nagy

**Affiliations:** Department of Plant Pathology, University of KentuckyLexington, KY, USA

**Keywords:** innate immunity, antiviral response, cell-intrinsic restriction factor, inhibition of virus replication, genome-wide screens, RNA-protein interaction, protein-protein interaction, protein network

## Abstract

To combat viral infections, plants possess innate and adaptive immune pathways, such as RNA silencing, R gene and recessive gene-mediated resistance mechanisms. However, it is likely that additional cell-intrinsic restriction factors (CIRF) are also involved in limiting plant virus replication. This review discusses novel CIRFs with antiviral functions, many of them RNA-binding proteins or affecting the RNA binding activities of viral replication proteins. The CIRFs against tombusviruses have been identified in yeast (*Saccharomyces cerevisiae*), which is developed as an advanced model organism. Grouping of the identified CIRFs based on their known cellular functions and subcellular localization in yeast reveals that TBSV replication is limited by a wide variety of host gene functions. Yeast proteins with the highest connectivity in the network map include the well-characterized Xrn1p 5′–3′ exoribonuclease, Act1p actin protein and Cse4p centromere protein. The protein network map also reveals an important interplay between the pro-viral Hsp70 cellular chaperone and the antiviral co-chaperones, and possibly key roles for the ribosomal or ribosome-associated factors. We discuss the antiviral functions of selected CIRFs, such as the RNA binding nucleolin, ribonucleases, WW-domain proteins, single- and multi-domain cyclophilins, TPR-domain co-chaperones and cellular ion pumps. These restriction factors frequently target the RNA-binding region in the viral replication proteins, thus interfering with the recruitment of the viral RNA for replication and the assembly of the membrane-bound viral replicase. Although many of the characterized CIRFs act directly against TBSV, we propose that the TPR-domain co-chaperones function as “guardians” of the cellular Hsp70 chaperone system, which is subverted efficiently by TBSV for viral replicase assembly in the absence of the TPR-domain co-chaperones.

Viruses with RNA genomes are widespread pathogens of plants, animals and humans. RNA viruses have small genomes with limited coding potential, yet they replicate efficiently in the infected host cells by co-opting numerous host proteins and subverting subcellular membranes to build replication factories/organelles (den Boon et al., 2010; Belov and van Kuppeveld, 2012; Nagy and Pogany, 2012; de Castro et al., 2013). During the infection process, RNA viruses rewire many cellular pathways that render the cells dramatically different from uninfected cells. However, cells have also developed antiviral strategies to limit viral infections and host organisms are in constant evolutionary battle with viruses, leading to several layers of antiviral responses from the host and emergence of novel suppressors/effectors by viruses.

Plants do not have the immune system of mammals or the potent interferon response, yet they possess innate immune pathways that provide non-specific and immediate response against bacteria, fungi, and viruses. In plants, the innate immune response involves pathogen-associated molecular pattern-triggered immunity (PTI), effector-triggered immunity (ETI), and different protein kinases that perform surveillance, systemic signaling and chromosomal changes (Jones and Dangl, 2006; Carr et al., 2010; Dangl et al., 2013). Adaptive immunity in plants is an inducible defense system that responds to environmental cues. Examples for adaptive immunity include the local hypersensitive response (HR) and systemic acquired resistance (SAR) response, which causes resistance at the whole-plant level (Durrant and Dong, 2004; Dempsey and Klessig, 2012).

The most potent adaptive immune response against plant viruses is based on RNA interference/RNA silencing (also called post-transcriptional gene silencing, PTGS), when the accumulating viral dsRNA formed during positive-stranded (+)RNA virus replication (Kovalev et al., 2014) induces the antiviral RNA silencing response (Ding, 2010; Pumplin and Voinnet, 2013; Szittya and Burgyan, 2013). Plant viruses counteract the RNA silencing pathways by using viral suppressors (Wu et al., 2010; Pumplin and Voinnet, 2013). Another type of innate resistance mechanism against plant viruses is based on R genes (dominant resistance), such as the N gene of tobacco against *Tobacco mosaic virus* (TMV) (Soosaar et al., 2005; Moffett, 2009). The N gene is a TIR-NB-LRR receptor, which recognizes the TMV helicase protein to induce plant innate immunity (Burch-Smith et al., 2007). A less well-understood innate resistance mechanism against plant viruses is based on recessive resistance genes. This type of innate immunity is usually based on recessive mutation(s) in host genes that are co-opted by the virus, but could no longer support the need of the virus during infection due to the mutations rendering the host protein “antiviral” (Carr et al., 2010; Wang and Krishnaswamy, 2012). In spite of our growing knowledge on the innate immune responses in plants, it is likely that additional, not yet identified, cell-intrinsic restriction factors (CIRFs) are also involved in limiting plant virus infections. This review discusses the genome-wide identification and detailed characterization of CIRFs with antiviral functions based on the plant-infecting tombusviruses.

## Identification of novel cell-intrinsic restriction factors during viral infection based on yeast

Most RNA viruses of plants have small RNA genomes coding for 5–15 viral proteins only that are insufficient to support viral replication without subverted host factors, subcellular membranes and cellular metabolites, such as ribonucleotides and amino acids (Mine and Okuno, 2012; Nagy and Pogany, 2012). The genome-size limitation and the biology of the virus makes RNA viruses much more dependent on the host cells in comparison with other plant pathogens, such as fungi and bacteria. In addition, the entire infectious cycle of plant RNA viruses takes place inside the infected cells, thus making the viral RNAs more accessible to cellular antiviral factors that could destroy viral RNAs and viral proteins in the cytosol. However, the limited number of effectors expressed by viruses, their intracellular presence and “the stealth mode” of viral activities also mean that viruses could more readily avoid recognition by the host in comparison with other pathogens. Therefore, plant cells might need to deploy numerous CIRFs against viruses. The identification of the putative CIRFs could be accelerated by unbiased genome-wide screens in host plants, which have not yet been accomplished.

Although *Saccharomyces cerevisiae* (baker yeast) lacks well-known antiviral pathways, such as the adaptive immune system of mammals, the interferon response or other innate immunity systems including the RNAi pathway, yeast cells can still protect themselves against viruses (Wickner, 1996; Wickner et al., 2013). To discover if yeast codes for CIRFs against viruses, high throughput screens using yeast genomic libraries have been performed based on small plant viruses, such as *Brome mosaic virus* (BMV) and *Tomato bushy stunt virus* (TBSV) (Nagy and Pogany, 2010).

Yeast is a powerful surrogate host for some plant viruses to help researchers screen for CIRFs. This is due to the small genome (only ~6000 genes, with 75% of genes have assigned functions and ~50% of genes have human and/or plant orthologs), and available extensive strain libraries (Nagy et al., 2014). Moreover, yeast not only facilitates genome-wide studies, it is also helpful for validation of the identified cellular factors and dissection of their functions to limit viral replication, as discussed below.

Although this review focuses on the results obtained mainly with TBSV, which is among the most intensively studied plant positive-strand (+)RNA viruses, CIRFs have also been identified for BMV and Flock house virus (FHV) an insect virus, based on yeast screens, as well (Kushner et al., 2003; Gancarz et al., 2011; Hao et al., 2014). Therefore, it is likely that detailed studies on CIRFs will expand to BMV, FHV, and possibly more viruses using yeast as a model host.

### High-throughput genome-wide screens in yeast for systematic identification of cell-intrinsic restriction factors limiting viral replication

The most efficient approach to identify CIRFs is based on unbiased genome-wide screens that measure the level of virus replication (Nagy and Pogany, 2010, 2012; Nagy, 2011; Hao et al., 2014; Xu and Cherry, 2014; Yasunaga et al., 2014). However, this approach is not yet straightforward to perform with plants that have a large number of genes and show high level of gene- (or functional-) redundancy that makes it challenging for scientists to identify CIRFs. The Ahlquist lab has pioneered the use of yeast as a viral host and performed a low throughput genetic mutagenesis screen and systematic screens to identify host genes affecting virus replication (Janda and Ahlquist, 1993; Kushner et al., 2003; Gancarz et al., 2011). The most extensive genome-wide screens based on yeast libraries were performed with TBSV (Panavas et al., 2005; Serviene et al., 2005, 2006; Jiang et al., 2006; Nagy and Pogany, 2010, 2012; Nagy, 2011). These included knock-out and knock-down libraries and a temperature-sensitive (ts) mutant library of yeast (Li et al., 2011) for TBSV replication studies, leading to the identification of 73 yeast genes acting as CIRFs against viral infection (Panavas et al., 2005; Serviene et al., 2005, 2006; Jiang et al., 2006; Shah Nawaz-Ul-Rehman et al., 2012, 2013).

**Figure 1:**
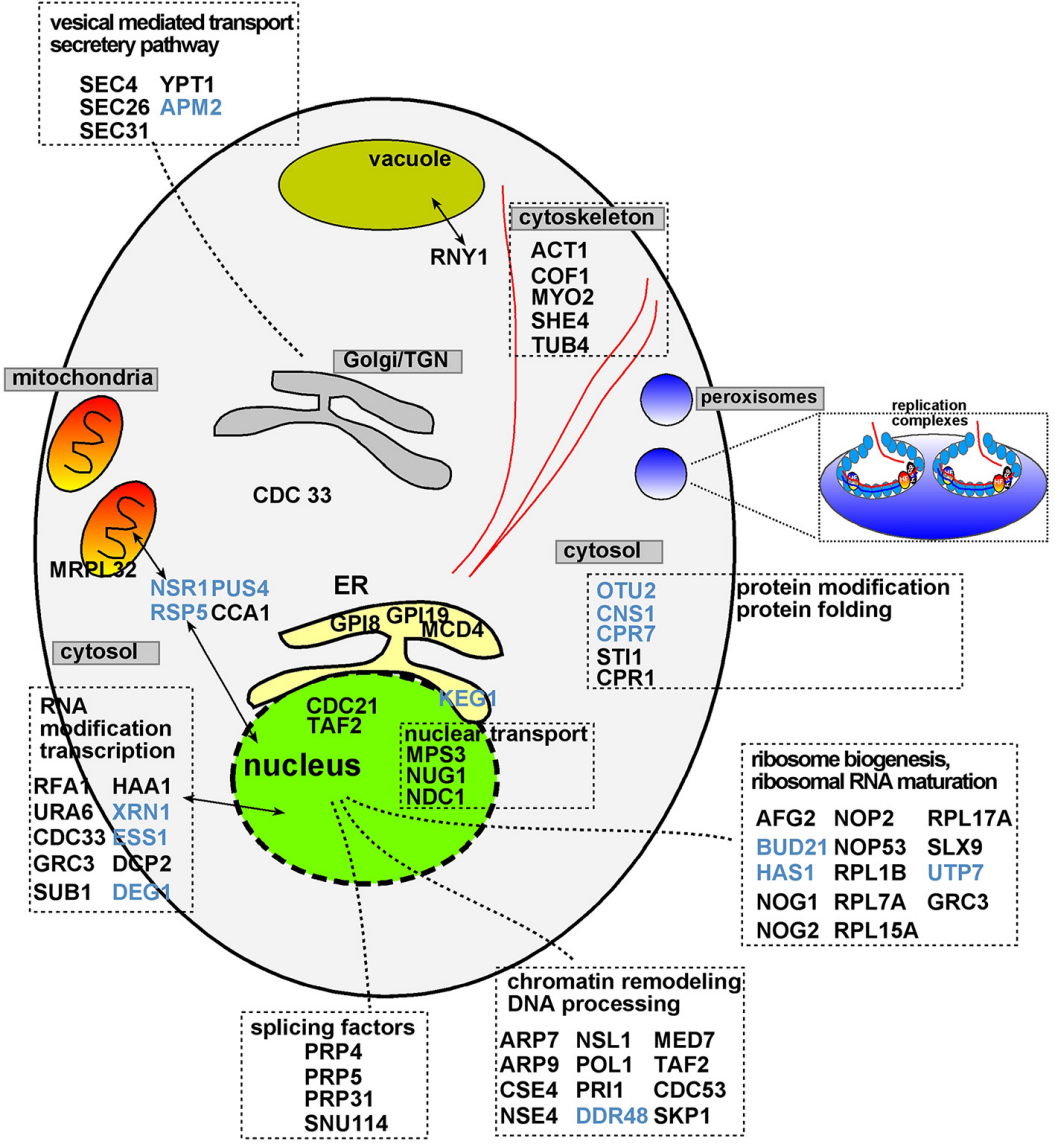
**Functions and subcellular localizations of cell-intrinsic restriction factors inhibiting tombusvirus replication in yeast**. Those cellular restriction factors that interact with the tombusvirus replication proteins or the viral RNA are shown in blue. Proteins present in two different subcellular compartments are shown with double-headed arrows. Note that TBSV utilizes the peroxisomal membranes for replication. The scheme also shows the ultrastructure of the tombusvirus VRCs as invaginations in the subcellular membranes.

Grouping of the identified CIRFs based on their known cellular functions and subcellular localization in yeast reveals that TBSV replication is limited by a wide variety of gene functions (Table 1). The largest groups among the CIRFs have functions related to RNA metabolism/processing/maturation, with 23 members involved in RNA processing and ribosome maturation and 8 proteins involved in RNA modification and additional 4 in RNA splicing (Figure 1). Another large group has 14 members involved in protein folding and modification/ubiquitination. Interestingly, 6 CIRFs are either part of the cytoskeleton or associated with it. Four secretory pathway proteins and two proteins involved in pyrimidine biosynthesis also showed CIRF activity against TBSV in yeast. The most unexpected groups of cellular proteins, which suppress TBSV replication, function in chromatin remodeling, transcription or nuclear transport (in all, 14 cellular proteins). We speculate that these last groups of proteins could possibly regulate the expression of direct antiviral factors or orchestrate the robustness of cellular antiviral responses. The enrichment of anti-TBSV cellular proteins with known nuclear functions is reminiscent of the findings with West Nile virus (WNV) based on RNAi screen in Drosophila cells (Yasunaga et al., 2014).

**Table 1:** **List of yeast CIRFs identified for tombusviruses based on high throughput yeast screens**.

**Name**	**Function**	**Localization**	**Plant ortholog**
ACT1	Actin	Cytoskeleton	AT3G12110/ACT11
AFG2	60S ribosomal biogenesis	Preribosome	–
APM2	Vesicle mediated transport	Vesicle transport	–
AQY1	Spore-specific water channel	pm	AT1G01620/PIP1C
ARP7	Chromatin remodeling, transcription regulation, DNA processing	SWI/SNF complex	–
ARP9	Chromatin remodeling, transcription regulation, DNA processing	SWI/SNF complex	–
BUD21	Component of small ribosomal subunit	Small ribosome	–
CCA1	Nucleotidyltransferase	mit, cyt, nuc	–
CDC21	Pyrimidine biosynthesis	Nucleus	AT4G34570/THY-2
CDC33	CAP-dependent mRNA translation initiation	Nucleus, cytoplasm	AT4G18040/EIF4E
CDC53	Involved in protein catabolic processes	Ubiquitin ligase complex (SCF)	AT1G26830/CUL3A
CNS1	Chaperons/co-chaperons, protein folding	cyt	AT1G04130/TPR2
COF1	Severs actin filaments	Cytoskeleton	AT2G31200/ADF6
CPR1	Chaperons/co-chaperons, protein folding	nuc, mit,	AT4G38740/ROC1
CPR7	Chaperons/co-chaperons, protein folding	cyt	–
CSE4	Chromatin accessibility and Pol II- binding regions	Nucleosome	–
DCP2	Decapping enzyme, and transcription initiation	Nucleus, cytoplasm	AT5G13570/DCP2
DDR48	DNA damage responsive protein	cyt	–
DEG1	Pseudouridine synthase	Nucleus, cytoplasm	AT1G34150
ESS1	Protein folding, chromatin silencing	nuc, cyt	AT2G18040
GPI19	Glycosylphosphatidylinositol synthesis	ER	–
GPI8	Glycosylphosphatidylinositol tranferase function	ER	AT1G08750
GRC3	Possibly involved in rRNA processing	nuc	–
HAA1	Transcriptional activator	Nucleus, cytoplasm	–
HAS1	RNA helicase, biogenesis of 40S, 60S ribosome subunits	nuc	AT5G65900
MCD4	Glycosylphosphatidylinositol synthesis	ER	–
MED7	Part of the Pol II mediator complex	nuc	AT5G03220
MPS3	Nuclear envelope/pore complex protein	Nuclear pore	–
MRPL32	Mitochondrial ribosomal protein	mit	–
MYO2	Actin based cargo transport	Cytoskeleton	AT5G43900/MYA2
NDC1	Subunit of the nuclear pore complex	Nuclear pore	–
NMT1	Myristoyl transferase	cyt	AT5G57020/ATNMT1
NOG1	60S ribosomal biogenesis	Preribosome	AT1G50920
NOG2	60S ribosomal biogenesis and nuclear export	Preribosome	AT1G52980/ATNUG2
NOP2	Processing and maturation of 27S pre-rRNA	Preribosome	AT5G55920/OLI2
NOP53	60S ribosomal biogenesis	nuc	AT2G40430
NSE4	DNA replication and repair	nuc	–
NSL1	MIND kinetochore complex	nuc	–
NSR1	Required for pre-rRNA processing	mit, cyt, nuc	AT1G48920/ATNUC-L1
NUG1	Nuclear export of the 60S ribosome	nuc	AT3G07050/NSN1
OTU2	Predicted cystein protease	cyt	AT3G62940
POL1	Required for DNA synthesis	nuc, mit	AT5G67100/ICU2
PRI1	Required for DNA synthesis	nuc	AT5G41880/POLA3
PRP31	Splicing factor	nuc	AT1G60170/EMB1220
PRP4	Splicing factor	snRNPcomplex	AT2G41500/LIS
PRP5	Prespliceosome formation	mit, cyt, nuc	–
PUS4	Pseudouridine synthase	mit, nuc	–
RFA1	DNA repair and replication	cyt, nuc	AT2G06510/ATRPA1A
RNY1	Vacuolar RNase, relocalizes to the cytosol upon stress	Vacuole, cytosol	AT2G02990/RNS1
RPL15A	Required for processing of pre-rRNA	Large ribosome	AT4G16720
RPL17A	Component of the 60S ribosomal subunit	Large ribosome	AT1G67430
RPL1B	Component of the 60S ribosomal subunit	Large ribosome	AT5G22440
RPL7A	Required for processing of pre-rRNA	Large ribosome	AT3G13580
RPT2	Proteasome component	nuc, proteasome	AT4G29040/RPT2a
RSP5	Ubiquitination	cyt, nuc, Golgi, pm	–
SEC26	Secretery pathway proteins	(COPI) coated vesicles	AT4G31480
SEC31	Secretery pathway proteins	(COPII) coated vesicles	AT3G63460/SEC31B
SEC4	Secretery pathway proteins	Actin cap, mit, vesicles, pm	AT3G09900/ATRABE1E
SHE4	Myosin function regulator	Cytoskeleton	–
SHO1	Transmembrane osmosensor	pm	–
SKP1	Part of the ubiquitin ligase complex (SCF)	nuc, cyt	AT5G42190/ASK2
SLX9	Pre-ribosomal RNA processing	Preribosome	–
SNU114	Splicing factor	nuc	–
STI1	Chaperons/co-chaperons, protein folding	cyt	AT4G12400/HOP3
SUB1	Transcriptional coactivator	nuc	–
TAF2	Pol II transcription initiation	TFIID complex nucleus	–
TUB4	Nucleates microtubules	Cytoskeleton	AT3G61650/TUBG1
URA6	Pyrimidine biosynthesis	nuc, cyt	AT5G26667/PYR6
UTP7	Processing of pre-18S rRNA	nuc	AT3G10530
XRN1	RNase, involved in ribosomal RNA maturation	Nucleus, cytoplasm	–
YPT1	Secretery pathway proteins	ER to Golgi vesicles, COPII coated vesicles, cyt vesicles, mit	AT1G02130/ATRAB1B

mit, mitochondria; cyt, cytoplasm; nuc, nucleus; pm, plasma membrane.

Those CIRFs that are underlined have direct physical interactions with viral components (RNA or replication proteins) based on prior proteomics screens.

As shown in Table S1, many of these yeast proteins have known orthologs in plants, suggesting conserved antiviral functions or pathways. Accordingly, we have shown for several plant orthologs to function as CIRFs against tombusviruses in plants (see below).

### Protein networks reveal high connectivity among many antiviral restriction factors

Since cellular proteins usually function with other proteins, it is possible that several of the identified CIRFs could be part of multiprotein complexes or members of particular cellular pathways and molecular networks. Indeed, many proteins have pleiotropic effects in cells, influencing the functions of several other proteins or cellular pathways. We speculate that some host proteins might inhibit virus replication indirectly through affecting the functions or availability of subverted pro-viral host factors, which directly interact with viral RNA or viral proteins.

Therefore, to gain insights into the functions of antiviral factors, we assembled protein networks including the identified CIRFs based on the yeast protein interaction map (SGD database, http://www.yeastgenome.org) (Cherry et al., 2012). We also included those subverted pro-viral host factors, which have been characterized in detail, into the interaction map. Finally, we also took advantage of our virus-host cell interactomes with viral replication protein-yeast protein and viral RNA-yeast protein maps based on global proteomics screens in yeast (Li et al., 2008, 2009; Mendu et al., 2010). These screens included (i) the analysis of the viral replicase complex via mass spectrometry approach (Serva and Nagy, 2006), (ii) viral RNA/viral replication proteins—host protein interactions based on a yeast protein array carrying ~4100 purified proteins that covers ~70% of yeast proteins (Li et al., 2008, 2009; Li and Nagy, 2011), and (iii) MYTH two-hybrid assay with yeast cDNA libraries (Mendu et al., 2010) leading to the construction of virus-host cell interactomes. These networks are expected to help identification of CIRFs with direct function against TBSV and those factors that might have more global effects on antiviral activities.

**Figure 2:**
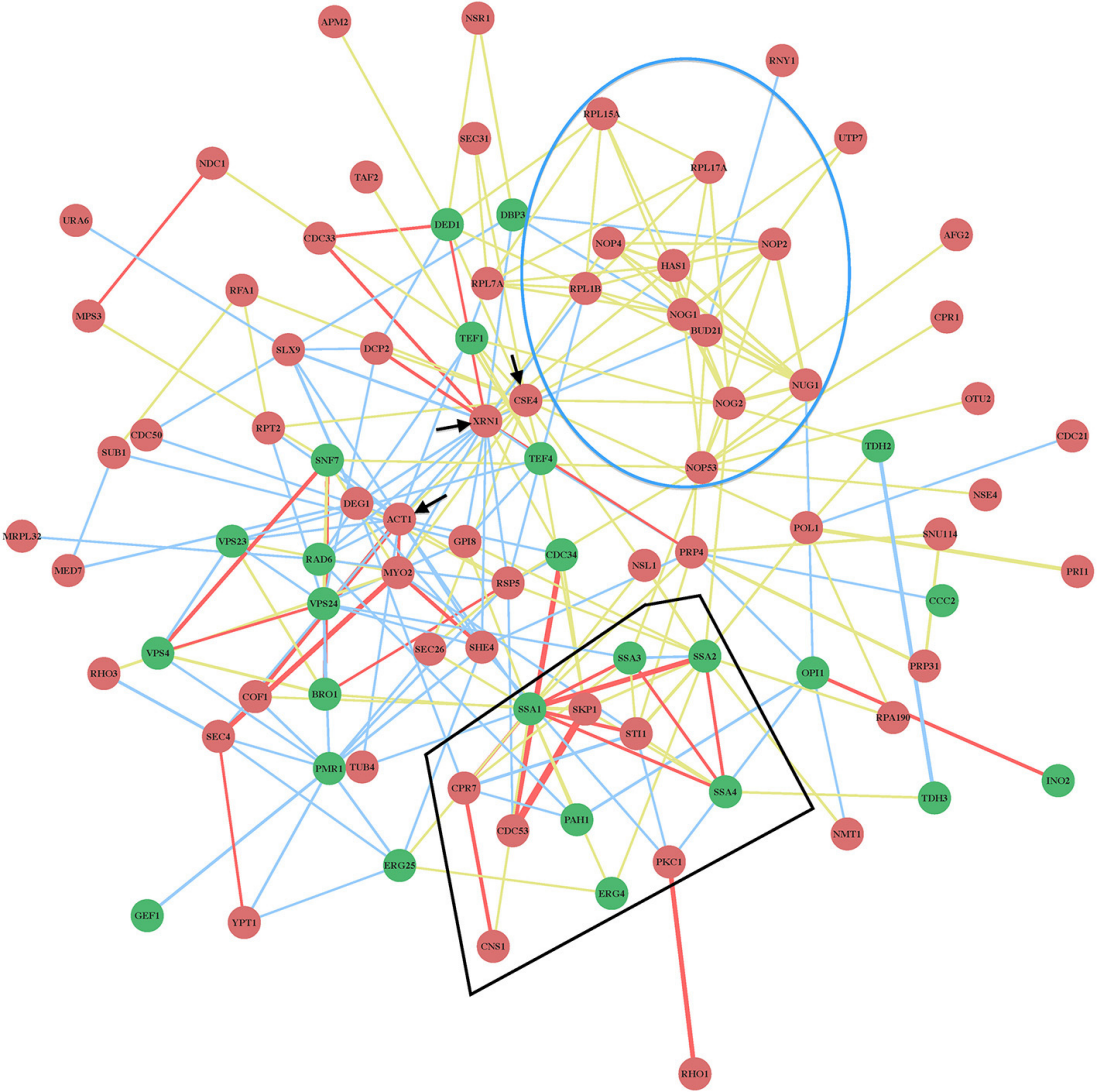
**Physical and genetic protein interaction networks of CIRFs and pro-viral host factors in yeast**. Functions of the genes are listed in Table 1. Red nodes indicate inhibitory CIRFs (i.e., viral replication goes up when the gene is deleted or down-regulated); Green nodes show positive pro-viral host factors (viral replication decreases when the gene is deleted or down-regulated); yellow lines indicate physical interactions; blue lines mark genetic interactions; red lines show both physical and genetic interactions. The thicker the line between two nodes, the greater the confidence of the interaction is. This means that there are more experimental data supporting the existence of the particular interaction. The blue circle encloses the largest group of related inhibitory factors with a similar function: biogenesis, processing and maturation of ribosomal structure, while the black polygonal lines indicate cellular factors, such as the TPR-domain co-chaperones, interacting with the Hsp70 (Ssa1-4) chaperone system. The black arrows mark the three proteins with the largest number of connections, namely, *XRN1, ACT1* and *CSE4* with 20, 17, and 14 connections, respectively. Note that *ARP7, ARP9, CCA1, DDR48, HAA1, MCD4, PRP5, and PUS4* genes (Table 1) are not included in the network map because they are not connected to the listed factors based on known interactions.

**Figure 3:**
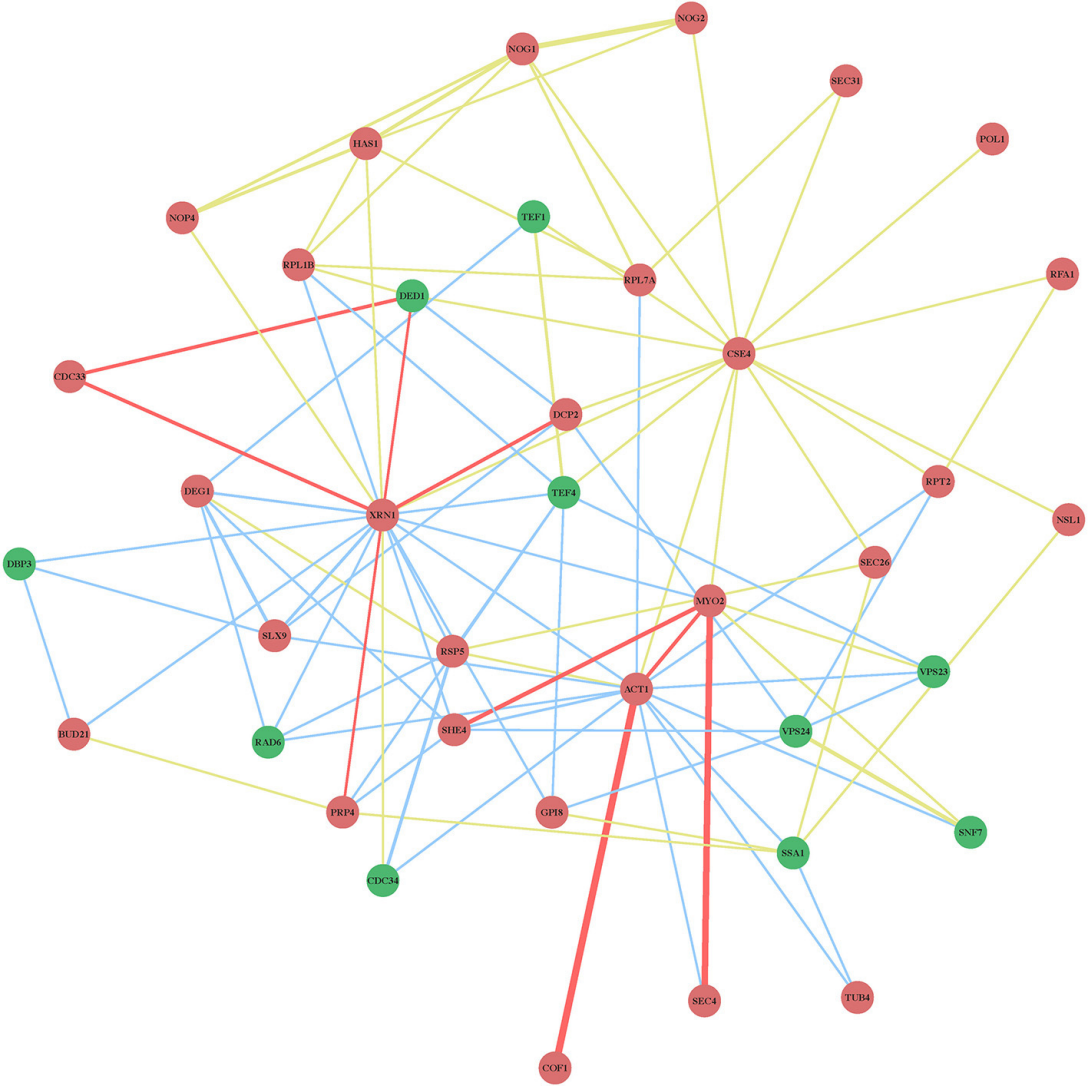
**Physical and genetic protein interaction networks including *XRN1, ACT1*, and *CSE4* CIRFs**. See further details in the legend to Figure 2.

The protein network map (Figure 2) with the previously identified cell-intrinsic TBSV restriction factors reveals several interesting observations. First, the yeast proteins with the highest connectivity in the network map include the well-characterized Xrn1p 5′–3′ exoribonuclease, and the not-yet characterized (as anti-TBSV proteins) Act1p actin protein and Cse4p centromere protein (Figure 2, marked with arrows and Figure 3). The protein network map also reveals an important interplay between the pro-viral Hsp70 cellular chaperone and the antiviral co-chaperones (Figure 2, see also below), and the ribosomal or ribosome-associated factors, whose antiviral activities have not yet been characterized in further details. These possibly key host proteins [the so-called hub proteins with high connectivity in cellular protein-protein interaction network (Tsai et al., 2009)] might target important viral components or host factors to inhibit TBSV replication. Interestingly, the protein network map excludes 8 CIRFs (Table 1). These factors might work as single antiviral factors, or their interactions map is not yet complete, thus leading to their omission from our protein network map (Figure 2).

## Characterization of antiviral functions of the identified cell-intrinsic restriction factors

Although the unbiased genome-wide screens are powerful to identify CIRFs, the actual antiviral functions of the identified cellular proteins are frequently obscure at the end of the screens. Therefore, detailed functional studies are required to dissect the mechanism of antiviral activities of given CIRFs as shown below with selected examples of host factors, such as the RNA binding nucleolin, ribonucleases, WW-domain proteins, single- and multi-domain cyclophilins, TPR-domain co-chaperones and cellular ion pumps.

### Exo- and endo-ribonucleases act as viral restriction factors

Cytoplasmic plant RNA viruses are controlled by the inducible RNAi pathway, which is an antiviral RNA degradation pathway in plants (Ding, 2010; Pumplin and Voinnet, 2013; Szittya and Burgyan, 2013). Yeast is lacking the RNAi machinery, thus favoring studies on antiviral effects of additional RNA degradation pathways. RNA viruses face cellular nucleases involved in general and specialized RNA degradation pathways. Therefore, not surprisingly, the accumulation of tombusvirus (+)RNA, which is non-capped at the 5′ end and lacks a 3′ poly(A) tail, is also greatly affected by nucleases in yeast. The best-characterized virus restriction factor is Xrn1p 5′–3′ exoribonuclease (Xrn4p in plants/mammals), which is involved in degradation of tombusvirus RNA, including partially degraded viral RNAs generated by endoribonucleases (Cheng et al., 2006, 2007; Jaag and Nagy, 2009). In the absence of Xrn1p in yeast or Xrn4 knockdown plants, tombusvirus RNA accumulation reaches several fold higher levels than in wt organisms. Cellular factors, such as Met22p bisphosphate-3′-nucleotidase, or LiCl/NaCl salt stress, which influence the activity of Xrn1p in cells also affect the accumulation of TBSV RNA (Jaag and Nagy, 2010), further strengthening the major function of Xrn1p as a CIRF against TBSV.

Unlike the potent Xrn1-based degradation pathway controlling TBSV accumulation in yeast, a less potent endoribonuclease-based TBSV RNA degradation pathway has also been documented in yeast, plants and *in vitro* (Jaag et al., 2011). This pathway includes the RNAse MRP (RNase mitochondrial RNA processing) complex with 10 proteins and one RNA component, leading to multiple internal cleavages within the tombusvirus (+)RNA. Interestingly, several of the internally-cleaved tombusvirus RNAs are still replication-competent and frequently participate in viral RNA recombination, generating virus sequence diversity in yeast and rapid emergence of new variants in plants (Jaag et al., 2011). However, the recombination process is also under the control of cellular Xrn1/Xrn4 (Jaag and Nagy, 2010). Thus, in addition to inhibiting tombusvirus RNA accumulation, Xrn1p/Xrn4p also affects the rate of virus evolution, suggesting complex interactions between host proteins and plant viruses (Nagy, 2008, 2011).

### Inhibitory functions of cellular RNA-binding proteins

Although cellular proteins involved in RNA processing/maturation/transport or ribosome biogenesis are the most abundant among the CIRFs against TBSV (Figure 1), currently only one of them is characterized in details. This is nucleolin (Nsr1p in yeast), which is a robust inhibitor of TBSV replication (Panavas et al., 2005). While deletion of *NSR1* in yeast leads to increased TBSV accumulation, over-expression of Nsr1p in yeast or the orthologous *Arabidopsis* nucleolin in *Nicotiana benthamiana* reduces the accumulation of tombusvirus RNA (Jiang et al., 2010). Moreover, addition of purified Nsr1p inhibits the *in vitro* replication of the tombusvirus RNA in a yeast-based cell free extract, suggesting a direct inhibitory function for nucleolin/Nsr1p. Accordingly, Nsr1p binds to the upstream portion of the 3′UTR in tombusvirus (+)RNA *in vitro*, which could lead to sequestration of the viral RNA, and inhibition of viral (+)RNA recruitment by the p33 replication protein for replication (Pogany et al., 2005; Jiang et al., 2010). The sequestration of the viral RNA occurs at the early stage of infection when the viral (+)RNA is present in limiting amounts.

The cellular nucleolin/Nsr1p is likely accessible to bind to the viral RNA in cells because Nucleolin/Nsr1p is an abundant, ubiquitously expressed protein that shuttles between the cytosol and the nucleus/nucleolus (Mongelard and Bouvet, 2007). Nucleolin is involved in ribosome biogenesis, in regulation of RNA polymerase I-based transcription, processing and modification of rRNA, proper folding of pre-rRNA, and nuclear—cytosolic transport of ribosomal subunits (Tuteja and Tuteja, 1998). Between the two nucleolin genes in *Arabidopsis*, only *AtNuc-L1* is expressed ubiquitously under normal growth conditions, and the plant nucleolin has similar functions to the yeast *NSR1* (Kojima et al., 2007; Pontvianne et al., 2007).

### WW-domain proteins function as cell-intrinsic restriction factors

**Figure 4:**
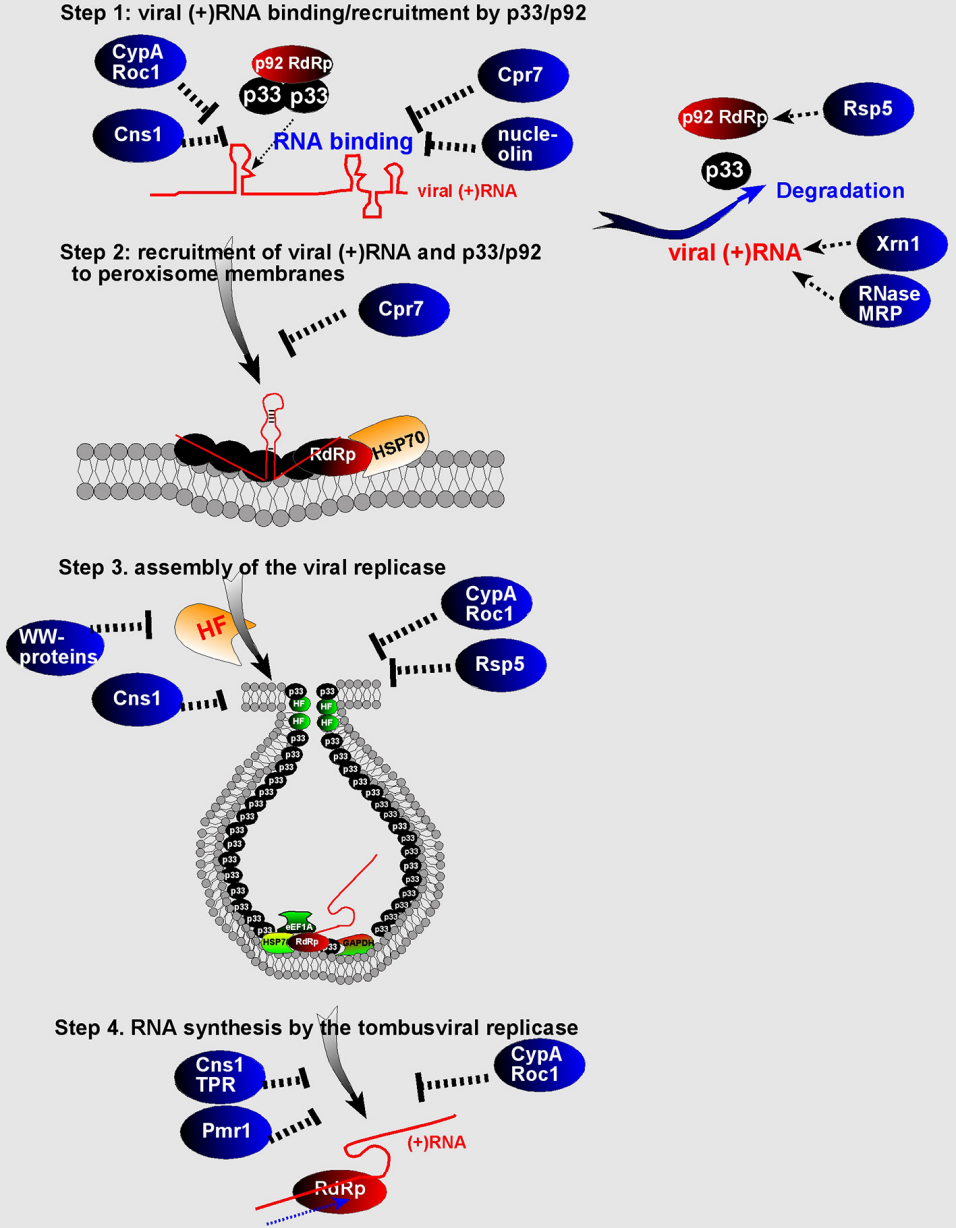
**Schematic presentation of the known or proposed roles of CIRFs in tombusvirus replication**. The four steps of the TBSV replication and degradation of viral components (p33/p92 and the viral RNA) are shown. “HF” indicates pro-viral host factors co-opted by TBSV. The virus induced spherule (vesicle-like structure) harboring the membrane-bound VRC is shown. See details in the text.

Functional studies with the yeast Nedd4-type Rsp5p E3 ubiquitin ligase revealed that Rsp5p binds to the tombusvirus replication proteins and inhibits tombusvirus replication in yeast (Barajas et al., 2009; Qin et al., 2012). Interestingly, the ubiquitin ligase function of Rsp5p was not critical for its inhibitory function, but its WW-domain containing three WW-motifs carried the antiviral activity. Moreover, binding of Rsp5p to the tombusvirus p92^pol^ replication protein leads to the degradation of p92^pol^ (Barajas et al., 2009; Qin et al., 2012). Expanding the research to additional WW-domain proteins has identified additional WW-domain proteins acting as CIRFs, such as the yeast Wwm1p and Prp40p and plant (*Arabidopsis*) AtDrh1, AtFCA, and AtPrp40c proteins (Qin et al., 2012). The activity of the purified TBSV replicase increased when purified from yeast with down-regulated expression of 4 WW-domain proteins, suggesting that WW-domain proteins are strong direct restriction factors of TBSV replication. Based on current data, it is predicted that the WW-domain proteins are strong inhibitors of TBSV replication by acting as competitors against co-opted host proteins for their recruitment into VRCs. In addition, WW-domain proteins might also directly inhibit the assembly of the TBSV VRCs by interacting with p33/p92^pol^ replication proteins and also promote the degradation of p92^pol^ (Figure 4).

The WW-domain is a simple and highly conserved protein domain involved in many protein-protein interactions (Macias et al., 2000; Hesselberth et al., 2006). The WW-domain contains two signature tryptophan residues and a conserved proline residue, which are part of a globular fold with three beta-sheets. WW-domain proteins bind to protein ligands carrying proline-rich sequences (Hesselberth et al., 2006). The WW-domain is present in many proteins and the WW-domain proteins function in protein trafficking, protein stability, apoptosis, and receptor signaling (Hesselberth et al., 2006; Salah et al., 2012). Interestingly, several WW-domain proteins have been identified in high throughput screens with a few RNA viruses, but their actual functions have not yet been studied. Overall, it seems that WW-domain proteins are very suitable for viral restriction functions, since they are present in the cytosol of all eukaryotic cells, and they also represent an ancient, very simple motif selected for protein: protein interactions (Hesselberth et al., 2006; Araya et al., 2012; Salah et al., 2012).

It is interesting to note that ubiquitination-based proteosomal degradation is likely function as CIRF against tymoviruses by destabilizing the viral RNA-dependent RNA polymerase during infection (Camborde et al., 2010; Chenon et al., 2012). In addition, another cellular degradation pathway that is based on autophagy seems to act as CIRF against several plant viruses (Liu et al., 2005; Derrien et al., 2012; Nakahara et al., 2012).

### Regulatory function of cellular Ca^2+^/Mn^2+^ ion pump protein in tombusvirus replication

A truly intriguing finding is the key role of the host Pmr1p intracellular Ca^2+^/Mn^2+^ ion pump in regulation of the efficiency of TBSV replication (Serviene et al., 2005; Jaag et al., 2010). Inactivation of *PMR1*, which codes for an ATPase-driven Ca^2+^/Mn^2+^ pump in yeast, leads to higher viral RNA accumulation, suggesting that Pmr1p is a CIRF. Pmr1p controls Ca^2+^ and Mn^2+^ influx to the Golgi from the cytosol in yeast cells (Ton and Rao, 2004). Research based on separation-of-function mutants of Pmr1p and cell-free TBSV replication assay revealed that the ability of Pmr1p to regulate the Mn^2+^ concentration in the cytosol is a key determinant of TBSV replication. Mechanistic approaches revealed that elevated Mn^2+^ level in yeast lacking Pmr1p enhances both TBSV replication and viral RNA recombinant accumulation due to the “super active” mode of operation for the tombusvirus replicase in the presence of high Mn^2+^ level (Jaag et al., 2010). The tombusvirus replicase utilizes Mg^2+^ ions in wild type yeast or plants that have low levels of Mn^2+^ in the cytosol, where TBSV replication takes place. Knockdown of *LCA1* and *ECA3* Ca^2+^/Mn^2+^ exporters in plants also leads to robust virus replication and RNA recombination, confirming that similar role of Mn^2+^ in regulation of TBSV replication exists in plants (Jaag et al., 2010). Thus, the intracellular environment/milieu in the vicinity of the viral replicase is also a key factor in viral replication.

### The cyclophilin superfamily members block the functions of tombusvirus replication proteins

A protein interaction screen with TBSV p33 replication protein has led to the identification of the yeast cyclophilin A-like Cpr1p (Cyclosporin A-sensitive proline rotamase 1) (Mendu et al., 2010). Since the single-domain Cpr1p is a member of the large cyclophilin (Cyp) family of proteins, additional studies identified other members of the Cyp family, including the cytosolic Cpr6p, Cpr7p, and Fpr1p as well as the mitochondrial Cpr3p that interacted with the TBSV p33 in yeast. Interestingly, several Cyps, such as yeast Cpr1p and Cpr7p and the plant Roc1 and Roc2 and the yeast Ess1p parvulin, efficiently inhibit tombusvirus replication in yeast and plants (Mendu et al., 2010; Lin et al., 2012). Since CypA and the orthologous *Arabidopsis* Roc1 and Roc2 cyclophilins inhibit TBSV replication in a cell free assay (Kovalev and Nagy, 2013), these proteins seem to act as direct CIRFs against TBSV.

Functional studies revealed that CypA binds to the RNA-binding domain of tombusvirus p33 replication protein and also to the viral (+)RNA. The published data support the model that binding of CypA and the plant orthologs to these viral components blocks the functions of the viral replication proteins in viral (+)RNA recruitment and VRC assembly (Figure 4) (Kovalev and Nagy, 2013).

Cyclophilins are a ubiquitous, highly conserved protein family with prolyl isomerase (PPIase) activity. Cyclophilins, the FKB proteins (FK506-binding proteins) and parvulins include 13 and 29 prolyl isomerases in yeast and in plants, respectively (Romano et al., 2004; Wang and Heitman, 2005). Cyclophilins share a 109 aa cyclophilin-like (CLD) domain surrounded by unique domains in each member of the family. Cyclophilins catalyze *cis*-*trans* isomerization of the peptidyl-prolyl bonds and are involved in the assembly of multidomain proteins, and in protein refolding after trafficking through cellular membranes, thus altering the structure, function or localization of the so-called client proteins (Arevalo-Rodriguez et al., 2004; Romano et al., 2004; Wang and Heitman, 2005). Altogether, PPIases play a global role in facilitating protein conformational changes and activation (Arevalo-Rodriguez et al., 2004). Cyclophilin expression is induced by biotic and abiotic stresses including salt stress, heat and cold shock, wounding, viral and fungal infections (Romano et al., 2004; Kumari et al., 2013).

Cyclophilins also inhibit other viral infections, such as influenza A virus and HIV-1 (human immunodeficiency virus-1) by binding and interfering with the nuclear localization of the influenza matrix protein (M1) (Liu et al., 2009). CypA gets incorporated into HIV-1 virions and inhibits the function of the viral Gag, the polyprotein precursor of virion structural proteins (Luban et al., 1993; Franke et al., 1994; Strebel et al., 2009). Interestingly, the retroviral Vif protein inhibits the incorporation of CypA into HIV particles, thus neutralizing the antiviral function of CypA (Takeuchi et al., 2007). WNV infection and FHV replication is also inhibited by cellular Cyps based on genome-wide screens for host factors (Krishnan et al., 2008; Hao et al., 2014). It is important to note that cellular cyclophilins could also facilitate virus replication, as seen with hepatitis C virus (HCV) (Gaither et al., 2010; Yang et al., 2010).

### Several TPR-domain proteins function as cell-intrinsic restriction factors

Detailed functional studies on the inhibitory roles of cellular cyclophilins on TBSV replication revealed that the TPR (tetratricopeptide repeats) domain in the cytosolic multi-domain (Cyp40-like) Cpr7p is a strong inhibitor of TBSV replication in yeast and *in vitro* (Lin et al., 2012). This discovery led to identification of the anti-tombusviral activities of additional TPR-domain proteins, such as Ttc4 oncogene-like Cns1p co-chaperone and the Hop/Sti1 co-chaperone, both of which bind to tombusvirus replication proteins (Lin and Nagy, 2013; Xu et al., 2014) and inhibit the p33/p92-driven recruitment of the TBSV RNA for replication and decrease the efficiency of VRC assembly (Lin et al., 2012). Thus, these co-chaperones with TPR-domains seem to act as direct CIRFs against tombusviruses.

Based on the known highly specific recognition of ligand proteins by TPR-domain proteins within the crowded cytosol (D'Andrea and Regan, 2003), we propose that the TPR-fold serves as a direct interaction platform with the tombusvirus replication proteins, blocking the functions of critical domains in the viral replication proteins, such as the RNA-binding or protein:protein interaction domains, needed for oligomerization and VRCs formation.

The TPR domains are highly variable, but they have common features, such as sharing 34 amino acid sequence repeats and showing a pattern of small and large hydrophobic amino acids that could adopt to a right-handed helical helix-loop-helix structure with an amphipathic channel (D'Andrea and Regan, 2003; Allan and Ratajczak, 2011). The TPR-domain proteins, which are involved in many protein-protein interactions, are abundant with 29 proteins in yeast (Haslbeck et al., 2013). TPR-domain proteins function in protein trafficking and protein import to organelles, apoptosis and synaptic vesicle fusion (Stawowczyk et al., 2011; Xiol et al., 2012). Interestingly, a few TPR-domain proteins have been shown to affect Chikungunya virus, WNV, Vesicular stomatitis virus, herpes simplex virus, poxvirus, and baculovirus infections (Daffis et al., 2010; Jeshtadi et al., 2010; Bourai et al., 2012; Danquah et al., 2012; Fensterl et al., 2012; Miettinen et al., 2012). TPR-domain proteins are also important in interferon-induced antiviral responses, and they help the antiviral activity of the IFIT protein family (Daffis et al., 2010; Liu et al., 2011; Pichlmair et al., 2011; Iki et al., 2012; Diamond and Farzan, 2013). Overall, many TPR-domain proteins could be suitable for viral restriction functions, since they are present in the cytosol, and they also represent an ancient, simple motif selected for protein:protein interactions (D'Andrea and Regan, 2003; Allan and Ratajczak, 2011).

### Are TPR-domain co-chaperones guardians of the Hsp70 chaperone system?

One of the emerging common themes in RNA virus replication is the hijacking of host cytosolic chaperones, such as Hsp70 and Hsp90, by various viruses (Tomita et al., 2003; Pogany et al., 2008; Wang et al., 2009; Weeks et al., 2010; Nagy et al., 2011). Subversion of cellular chaperones by RNA viruses could be an easy task for viruses, since Hsp70s have promiscuous protein recognition due to binding to short linear stretches of hydrophobic residues in protein substrates (Kampinga and Craig, 2010; Mayer, 2010; Taipale et al., 2010). In addition, RNA viruses produce large quantities of viral-coded proteins that need cellular chaperones for correct folding. Also, viruses co-opt host chaperones to regulate viral replication (Nagy et al., 2011). Furthermore, another advantage of subversion of cellular chaperones by viruses is that antiviral processes, such as cell signal transduction, depend on cellular chaperones. Thus, sequestering chaperones for viral functions could block antiviral responses by the cell and prevent premature cell death, thus creating a favorable microenvironment for virus replication.

**Figure 5:**
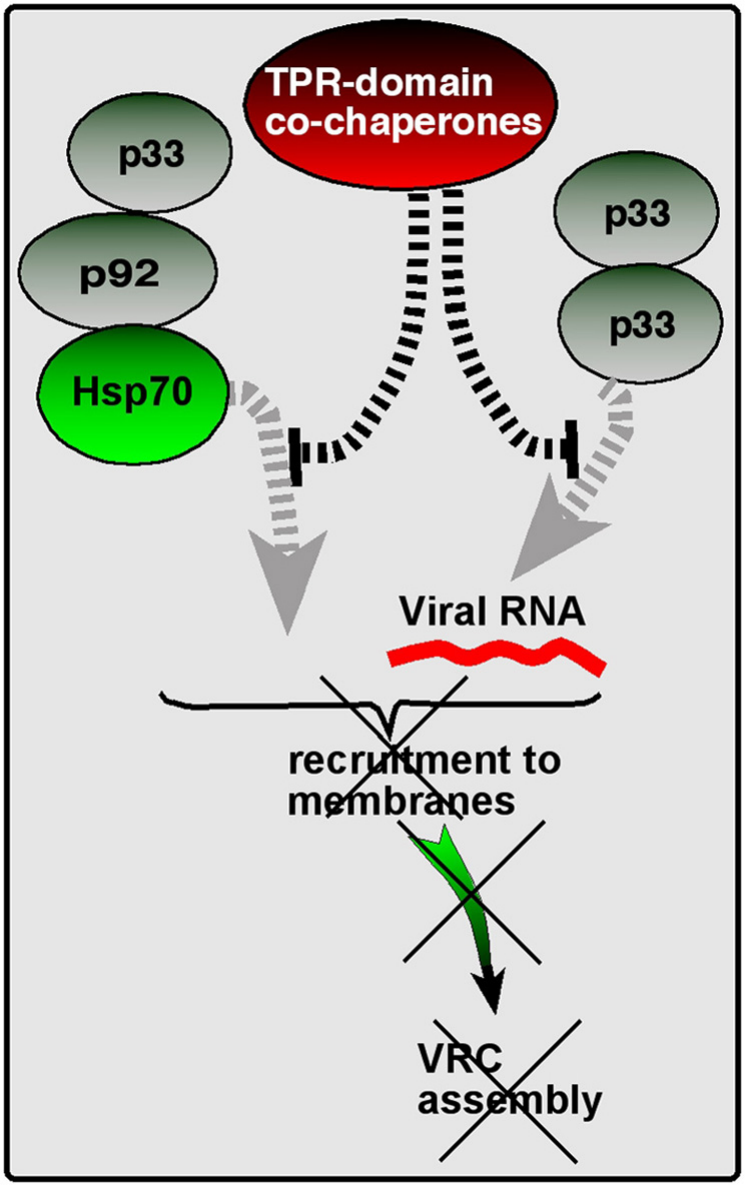
**A model of the “guardian of Hsp70” function of TPR-containing co-chaperones**. The ATP-dependent Hsp70 molecular chaperone is usurped by TBSV to facilitate recruitment of viral components to the subcellular (peroxisomal) membranes and also to promote VRC assembly. However, TPR-containing co-chaperones, such as the yeast Cyp40-like Cpr7p, Ttc4 oncogene-like Cns1p, and the Hop/Sti1 co-chaperones, might protect the Hsp70 chaperones from falling easy “prey” to TBSV by interacting with viral components. These events lead to the inhibition of viral processes, as shown, explaining how these co-chaperones work as CIRFs.

However, our discovery of the CIRF function of several TPR-domain co-chaperones, including the Cyp40-like Cpr7p, Ttc4 oncogene-like Cns1p, and the Hop/Sti1 co-chaperone suggest that these co-chaperones might protect the Hsp70/Hsp90 chaperones from falling easy “prey” to the envading viruses. These co-chaperones are conserved, highly abundant proteins lacking chaperone activity on their own, and form complexes with Hsp70 and Hsp90 chaperones and their clients (Wegele et al., 2003; Flom et al., 2007). They have three TPR domains, which are involved in binding to Hsp90s and Hsp70s and playing roles in client protein transfer from the Hsp70 complex to the Hsp90 complex (Odunuga et al., 2003; Schmid et al., 2012). Since these co-chaperones also bind to the tombusvirus replication proteins, we predict that the TPR-domain co-chaperone proteins help the host to prevent the hijacking/recruitment of Hsp70 and Hsp90 by tombusviruses to build VRCs (Figure 5). Therefore, these co-chaperones could be “guardians” of the Hsp70/Hsp90, thus performing cell-intrinsic antiviral activities.

## Cell-intrinsic restriction factors in plants

**Figure 6:**
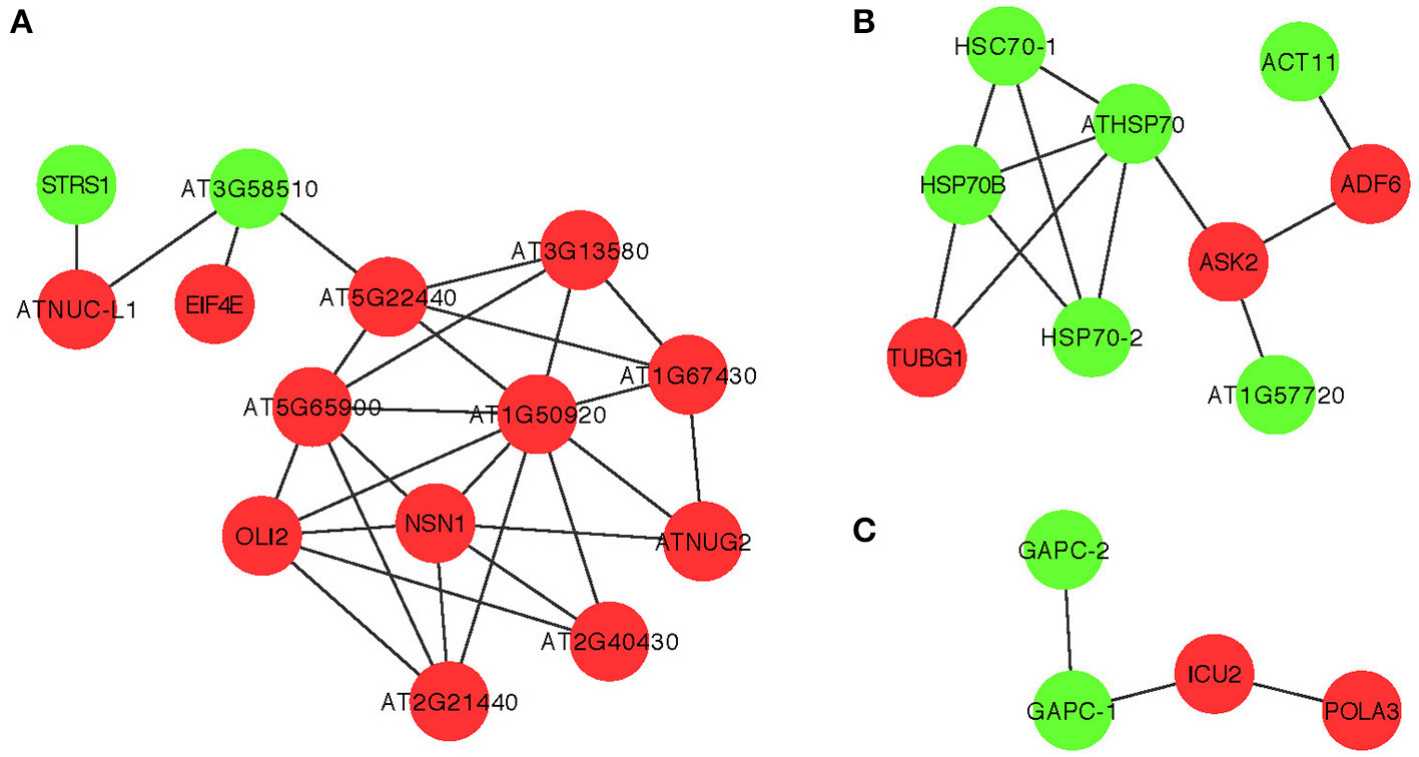
**Physical and genetic protein interaction networks of predicted orthologous cell-intrinsic restriction factors and pro-viral host factors in plants**. Functions of the *Arabidopsis* genes are listed in Table 1. Red nodes indicate inhibitory CIRFs, while green nodes show positive proviral host factors. **(A–C)** The three major groups of molecular networks with predicted orthologous CIRFs of *Arabidopsis*. These three small networks were generated with *Arabidopsis thaliana* orthologs to represent the most important interactions common to *S. cerevisiae* and *A. thaliana.* In panel **(A)**, the *Arabidopsis* orthologs include *DBP3* (STRS1), *DED1* (AT3G58510), *NSR1* (ATNUC-L), and *CDC33* (EIF4E), panel **(B)** shows the conserved interactions between Hsp70 family proteins and *ACT1* (ACT11), *TUB4* (TUBG1), *COF1* (ADF6), and *SKP1* (ASK2) orthologs, while panel **(C)** indicates the conserved interactions between *GAPDH* genes and *POL1* (ICU2) and *PRI1* (POLA3) orhologs.

Although it is not yet feasible to perform systematic genome-wide screens in plants—similar to those screens in yeast (see above)—it is critical to study the antiviral functions of the identified CIRFs in the native plant hosts. This is possible because of the high functional conservation of many cellular factors from yeast to plants to animals. Indeed, many of the identified yeast CIRFs against tombusviruses have known orthologs in plants, suggesting conserved antiviral functions or pathways. Accordingly, we have shown for ~10 plant orthologs that they function as CIRFs against tombusviruses using *Nicotiana benthamiana* plants. In addition, protein network analysis of orthologous plant genes with putative CIRF functions also showed three highly-connected protein groups, such as the ribosomal proteins, the Hsp70 network and nuclear/transcription factors (Figure 6), similar to those observed with the yeast proteins (Figure 2).

The validation experiments were based on knocking down host protein levels by a virus-induced gene silencing approach. Alternatively, over-expression of the wt or dominant-negative mutant of a given host protein could also be used in the natural system to confirm the antiviral effect. Altogether, the validation experiments in the natural hosts are important to measure the antiviral potential of CIRFs. The natural host also allows testing the effect of the identified CIRFs, which act against tombusviruses, on other related or even unrelated viruses to estimate how broad the antiviral effect could be.

## Concluding remarks and future perspectives

Plants use multiple layers of defense against cytosolic plant viruses. In addition to the inducible RNA silencing pathway, plants also have dominant and recessive resistance genes against selected plant viruses that trigger antiviral responses. A recently emerging concept in innate immunity is the presence of numerous CIRFs in host genomes that greatly reduce plant virus replication and likely facilitate combating viruses and making the above induced and passive innate immune responses more potent.

Systematic genome-wide screens using yeast as a model host have allowed for the identification of more than 70 CIRFs against tombusviruses. Many of the identified yeast CIRFs are conserved in plants and, accordingly, ~10 CIRFs of tombusviruses have already been characterized in plants. The three major sets of CIRFs seem to have either (i) direct inhibitory effect on tombusvirus replication by blocking the functions of the tombusvirus components or (ii) protecting (guarding) host components, such as the Hsp70/Hsp90 chaperones systems, from being efficiently hijacked by tombusviruses; or (iii) more global effects on antiviral activities by regulating the expression of direct antiviral factors or orchestrating the robustness of cellular antiviral responses.

### Conflict of interest statement

The authors declare that the research was conducted in the absence of any commercial or financial relationships that could be construed as a potential conflict of interest.
