# Supplementary material for: Tombusvirus-yeast interactions identify conserved cell-intrinsic viral restriction factors
Source: Front Plant Sci. 2014 Aug 11;5:383. doi: 10.3389/fpls.2014.00383 (PMC4127529; doi:10.3389/fpls.2014.00383)

Supplementary materials:

MATERIALS AND METHODS

Bionformatical analysis of the interactions between inhibitory cell-intrinsic restriction factors and pro-viral host factors.

For yeast genes: The function and the systematic name of each gene/protein in Fig. 2 or Table 1 were obtained from SGD database [Cherry, J.M., Hong, E.L., et al., 2012]. Later each gene/protein was analyzed using the BioGRID interaction database [Stark, C., Breitkreutz, B.J. et al., 2011] in order to find interactions among the positive pro-viral factors and the inhibitory factors obtained from previous genome-wide screens with TBSV. Results were deposited in a plain text format file that was used afterwards as input to a program written in the R programming language [R Core Team, 2014] that generates a PDF file with a network depicting the found interactions. Parameters like type of interaction (physical, genetic or both) and confidence (number of experiments that support the interaction) were considered in the program. The code of the program requires the previous installation of the "igraph" package [Csardi, G. and Nepusz, T., 2006] particularly from the CRAN mirror USA CA-2 in order to run properly. For *A. thaliana* orthologs: First of all a search was done to find *Arabidopsis* orthologs of the yeast genes in Fig. 6 or Table 1 using InParanoid database [Berglund, A.C., Sjolund, E., et al., 2008]; it was necessary to use the yeast gene systematic name and limit the search to *A. thaliana* species. We chose only the *A. thaliana* loci that have a score between 0.9 and 1. Subsequently we looked for the conserved interactions, this mean the ones found between the yeast genes/proteins and also between the *Arabidopsis* orthologs of that same yeast genes/proteins. Using the acquired information three small networks were free-hand drawn with Cytoscape software [Shannon, P., Markiel, A., et al., 2003] representing the largest gene groups with conserved interactions in yeast and plant.

REFERENCES

Berglund, A.C., Sjolund, E., Ostlund, G., and Sonnhammer, E.L. (2008). InParanoid 6: eukaryotic ortholog clusters with inparalogs. *Nucleic Acids Res*. 36:D263-266

Cherry, J.M., Hong, E.L., Amundsen, C., Balakrishnan, R., Binkley, G., Chan, E.T., Christie, K.R., Costanzo, M.C., Dwight, S.S., Engel, S.R., et al. (2012) Saccharomyces Genome Database: the genomics resource of budding yeast. *Nucleic Acids Res*. 40:D700–D705.

Csardi, G., and Nepusz, T. (2006). The igraph software package for complex network research. *InterJour Comp Sys*. 1695. http://igraph.org

R Core Team (2014). R: A language and environment for statistical computing. R Foundation for Statistical Computing, Vienna, Austria. URL http://www.R-project.org/.

Shannon, P., Markiel, A., Ozier, O., Baliga, N.S., Wang, J.T., Ramage, D., Amin, N., Schwikowski, B., Ideker, T. (2003). Cytoscape: a software environment for integrated models of biomolecular interaction networks. *Genome Res*. 13:2498–2504.

Stark, C., Breitkreutz, B.J., Chatr-Aryamontri, A., Boucher, L., Oughtred, R., Livstone, M.S., Nixon, J., Van Auken, K., Wang, X., Shi, X., Reguly, T., Rust, J.M., Winter, A., Dolinski, K., Tyers, M. (2011) The BioGRID Interaction Database: 2011 update. *Nucleic Acids Res.* 39: D698–D704.

Table S1. List of yeast CIRFs and the plant orthologs.

GENE NAMES	At ORTHOLOG	Nt ORTHOLOG Name/GI accession number	Sl ORTHOLOG Locus/GI accession number
ACT1/YFL039C	AT3G12110/ACT11	TOB66/50058114	LOC101264601/460378622
AFG2/YLR397C	-	DQ515925/98962496	LOC101266129/460391350
APM2/YHL019C	-	-	-
ARP7/YPR034W	-	-	-
ARP9/YMR033W	-	-	-
BUD21/YOR078W	-	-	-
CDC21/YOR074C	AT4G34570/THY-2	-	LOC101267455/460370058
CDC33/YOL139C	AT4G18040/EIF4E	-	-
CDC53/YDL132W	AT1G26830/CUL3A	-	-
COF1/YLL050C	AT2G31200/ADF6	ADF1/22857911	-
CSE4/YKL049C	-	-	LOC101250929/460405317
DCP2/YNL118C	AT5G13570/DCP2	-	-
DEG1/YFL001W	AT1G34150	-	-
GPI8/YDR331W	AT1G08750	-	LOC101249586/460405482
HAS1/YMR290C	AT5G65900	-	LOC101245343/460380720
MED7/YOL135C	AT5G03220	-	-
MPS3/YJL019W	-	-	-
MRLP32/YCR003W	-	-	-
MYO2/YOR326W	AT5G43900/MYA2	MY170/56201390	LOC101248616/460404667
NDC1/YML031W	-	-	-
NMT1/YLR195C	AT5G57020/ATNMT1	-	-
NOG1/YPL093W	AT1G50920	-	LOC101249658/460395603
NOG2/YNR053C	AT1G52980/ATNUG2	-	LOC101250712/460397315
NOP2/YNL061W	AT5G55920/OLI2	-	-
NOP4/YPL043W	AT2G21440	-	-
NOP53/YPL146C	AT2G40430	-	-
NSE4/YDL105W	-	-	-
NSL1/YPL233W	-	-	-
NSR1/YGR159C	AT1G48920/ATNUC-L1	-	-
NUG1/YER006W	AT3G07050/NSN1	-	LOC101252093/460393469
OTU2/YHL013C	AT3G62940	-	-
PKC1/YBL105C	-	Serine/threonine-protein kinase/608604526	LOC101261560/460408031
POL1/YNL102W	AT5G67100/ICU2	-	-
PRI1/YIR008C	AT5G41880/POLA3	-	LOC101258292/460399993
PRP4/YPR178W	AT2G41500/LIS	-	-
PRP31/YGR091W	AT1G60170/EMB1220	-	-
RFA1/YAR007C	AT2G06510/ATRPA1A	-	LOC101250913/460400799
RHO1/YPR165W	-	RAC4/27527522	LOC101251031/460367732
RHO3/YIL118W	-	RAC/6015626	LOC101248622/460406412
RNY1/YPL123C	AT2G02990/RNS1	-	-
RPA190/YOR341W	AT3G57660/NRPA1	RPB1/188532150	LOC101260591/460408654
RPL1B/YGL135W	AT5G22440	-	LOC101252594/460391426

RPL7A/YGL076C	AT3G13580	-	LOC101251486/460394399
RPL15A/YLR029C	AT4G16720	-	LOC101252296/460392626
RPL17A/YKL180W	AT1G67430	-	LOC101265739/460391858
RPT2/YDL007W	AT4G29040/RPT2A	RPT6/442539517	LOC101267617/460393571
RSP5/YER125W	-	-	LOC101249744/460396964
SEC4/YFL005W	AT3G09900/ATRABE1E	RAB8-1/18447912	LOC101264577/460408393
SEC26/YDR238C	AT4G31480	-	LOC101248531/460407938
SEC31/YDL195W	AT3G63460/SEC31B	-	-
SHE4/DIM1/YOR035C	-	-	-
SKP1/YDR328C	AT5G42190/ASK2	SKP1/51292006	LOC101268328/460370416
SLX9/YGR081C	-	-	-
SNU114/YKL173W	-	-	-
SUB1/YMR039C	-	-	-
TAF2/YCR042C	-	-	-
TUB4/YLR212C	AT3G61650/TUBG1	GTUB1/11414995	LOC101260712/460381165
URA6/YKL024C	AT5G26667/PYR6	-	-
UTP7/YER082C	AT3G10530	-	-
YPT1/YFL038C	AT1G02130/ARA5	X72212/311906	LOC101255135/460367759

At: Arabidopsis thaliana; Nt: Nicotiana tabacum (tobacco); Sl: Solanum lycopersicum (tomato)

Fig. S1. Physical and genetic protein interaction networks of cell-intrinsic restriction factors and pro-viral host factors in yeast. Gene names in yellow indicate genes that have plant orthologs in *Arabidopsis thaliana*, *Nicotiana tabacum* or *Solanum lycopersicum* as depicted in Table S1. Red nodes indicate inhibitory cell-intrinsic restriction factors (i.e., viral replication goes up when the gene is deleted or down-regulated); Green nodes show positive pro-viral host factors (viral replication decreases when the gene is deleted or down-regulated); yellow edges indicate physical interactions; blue lines mark genetic interactions; red lines show both physical and genetic interactions. The thicker the line between two nodes, the greater the confidence of the interaction is. This means that there are more experimental data supporting the existence of the particular interaction.

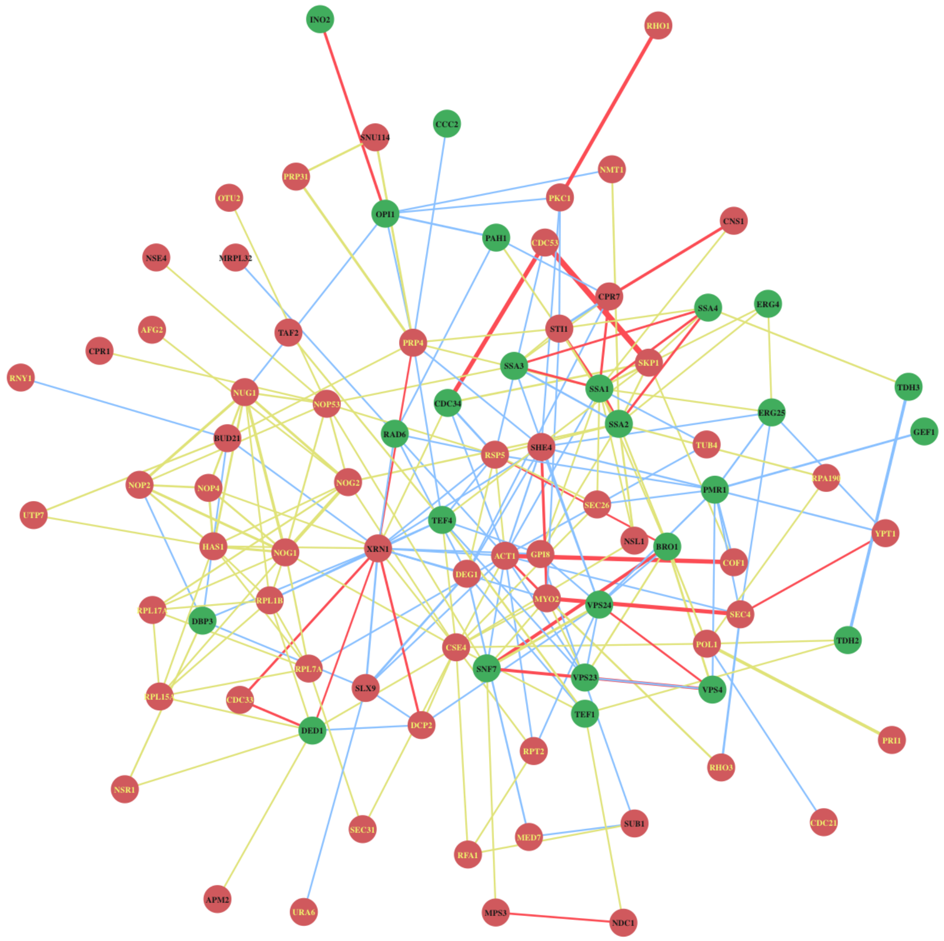

Supplement: Supplementary file 1 [file Presentation1.PDF]
